# Investigating the predictors of chronic care annual funding requirements under activity-based funding

**DOI:** 10.1186/1472-6963-15-S2-A3

**Published:** 2015-06-15

**Authors:** Steve Gillett, Kylie Houlihan, Wilf Williams

**Affiliations:** 1 SSAKG Consulting, Mulgrave, Victoria, 3170, Australia; 2 Queensland Health Department, Brisbane, Victoria, 4000, Australia; 3 KPMG, Brisbane, Queensland, 4000 Australia

## Background

Most health systems in developed countries are struggling to contain the costs of providing high-quality health care to their populations. Factors such as population growth, demographic aging, changing clinical practices, and lower mortality all result in increased demand for hospital services. In addition, falling mortality rates result in a larger number of people living with chronic conditions for a longer amount of time. The increased demand for services and the prevalence of chronic disease lead to higher health care costs.

Increasingly, countries are focusing on ways to keep people living with chronic conditions relatively healthy and being treated within the primary care setting — rather than allowing their condition to deteriorate until hospital care is required. Doing so requires implementing funding models that encourage integrated, effective, and preventive care.

As a first step in developing such alternative models, it is useful to understand the costs of providing care under the current model, and to be able to predict which individual patients might benefit under an alternative care model.

## Materials and methods

We examined hospital utilization data for patients admitted to a Queensland (Australia) public hospital between 2009-2010 and 2011-2012 who were reported, on at least one admission, to have a chronic disease diagnosis.

We estimated the funding associated with the chronic disease by assessing the impact of specific chronic ICD codes on the activity-based funding payments (National Weighted Activity Units) for a 12-month period following a patient’s first hospital admission with a reported chronic diagnoses. Adjustments were made for people who were not subsequently admitted in the 12-month period.

We used our simple model to stratify patients into risk categories based upon their predicted use of services. Actual annual hospital use was then compared between risk categories (described below).

## Results

Approximately 80% of patients were rated as relatively “low risk,” with only 2% rated as “extreme risk.” Members of the “extreme” group used, on average, four times the resources of the low-risk group.

We found that a combination of specific conditions, indigenous status, proximity to death, and remoteness all influenced not only funding requirements but also re-admission in the 12 months following the first admission. Age was not found to be a consistent factor after adjusting for these other variables.

Further, the specific parameter estimates appear to have face validity, with increasing funding requirements stemming from more complete combinations of chronic disease; this suggests the possibility of using a person’s previously documented illnesses to predict future care requirements.

## Conclusions

We describe a relatively simple way of integrating an annual payment model for chronic care within an activity-based funding structure. This methodology provides hospitals with a level of funding based upon the anticipated use of resources to treat individual patients’ chronic conditions over the year.

While this predictive power is currently modest, we demonstrate that a person’s previous admission history can be used to predict resource requirements and, potentially, identify patients for alternative care models. In presenting these findings we recognize and discuss the limitations of our study design and discuss possible ways forward.

